# Notch signaling in cancers: mechanism and potential therapy

**DOI:** 10.3389/fcell.2025.1542967

**Published:** 2025-02-20

**Authors:** Chen Chen, Yan Du, Rongzu Nie, Shuangshuang Wang, Hang Wang, Peifeng Li

**Affiliations:** ^1^ College of Food and Bioengineering, Zhengzhou University of Light Industry, Zhengzhou, China; ^2^ College of Food Science and Engineering, National Engineering Research Center of Wheat and Corn Further Processing, Henan University of Technology, Zhengzhou, China

**Keywords:** notch signaling, cancer, mechanism, mutations, potential therapy

## Abstract

The Notch signaling pathway is an evolutionarily conserved intercellular signaling cascade that regulates a number of cellular processes, including cell development, proliferation, apoptosis, and genome stability. The Notch signaling pathway is pervasive in the human body, affecting tumorigenesis and progression, which is one of the most significant signaling pathways in this regard, influencing various receptors and cellular functions of tumor cells. Aberrant expression or mutation of Notch has been linked to the onset and progression of a variety of malignant tumors. In this review, we discussed the mechanism of Notch signaling in lung, liver and colorectal cancer and explored future strategies and directions for cancer treatment by modifying the Notch signaling pathway.

## Introduction

In 1917, Morgan and his colleagues published the first description of a notch on the edge of the wing of *Drosophila*. This was the result of a heterozygous deletion of a gene on the X chromosome, which was subsequently designated “Notch” (Morgan, 1917). As a highly conserved intercellular signaling pathway, “Notch” was observed in a variety of eukaryotic organisms. It transmits signals through interactions between neighboring cells and can influence cell proliferation, differentiation, migration, growth, and apoptosis. The determination of cell fate was a crucial function of the Notch signaling pathway, which was critical in a multitude of biological processes, including growth, development, and tissue repair (Vijayaraghavan et al., 2018).

**FIGURE 1:**
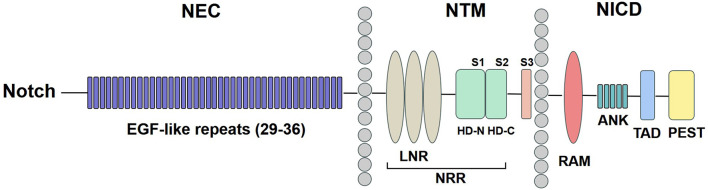
Notch receptor structure. The NEC region has 29–36 EGF-like repeats. The NTM region contains LNR and a HD domain followed by S1, S2, and S3. The NICD comprises a RAM domain, ANK repeats, a TAD, and a PEST domain. NEC: Notch extracellular; EGF: epidermal growth factor; NTM: Notch transmembrane; LNR: Lin12-Notch repeats; HD: heterodimerization; NRR: negative regulatory region; NICD: Notch intracellular domain; RAM: recombination signal-binding protein 1 for the J-kappa-association molecule; ANK: ankyrin; TAD: transactivation domain; PEST: proline/glutamine/serine/threonine-rich.

The Notch signaling pathway consists of five ligands: Delta-like 1, 3, 4 (Dll1, Dll3, Dll4) and Jagged 1, 2 (Jag1, Jag2) and four Notch receptors (Notch1, Notch 2, Notch3, Notch 4) (Kovall et al., 2017). The precursor of the Notch receptor protein is synthesized in the endoplasmic reticulum and subsequently cleaved by the Furin protease in the Golgi apparatus. This process generates a heterodimeric Notch protein, which is then transferred to the cell surface (Kopan and Ilagan, 2009). As shown in Figure 1, Notch receptors are type I transmembrane proteins, comprising Notch extracellular (NEC), Notch transmembrane (NTM), and Notch intracellular domain (NICD). The NEC domain is a heterodimeric protein composed of 29–36 epidermal growth factor (EGF)-like repeats, which facilitate binding to Notch ligands. Additionally, it contains three cysteine-rich repeats and a heterodimerization (HD) structural domain, which serve to block ligand-independent signaling. The NTM region contains Lin12-Notch repeats (LNR) and a HD domain followed by three cleavage site 1 (S1), S2, and S3. The NICD comprises a recombination signal-binding protein 1 for the J-kappa-association molecule (RAM) domain, ankyrin (ANK) repeats, a transactivation domain (TAD), and a proline/glutamine/serine/threonine-rich (PEST) domain (Zhou et al., 2022).

## Activation of the Notch signaling pathway

The interaction of Notch receptors with ligands in neighbouring cells leads to activation of the Notch signaling pathway, and this interaction is completed by a three-fold enzymatic cleavage process. Firstly, Notch receptor precursor proteins are synthesized in the endoplasmic reticulum. The newly synthesized Notch proteins are then glycosylated and cleaved for the first time in the Golgi apparatus by the furin protease converting enzyme at the S1, which is located about 70 amino acids outside the transmembrane segment, forming the NEC and NTM (Tyagi et al., 2020). These are subsequently bound together by noncovalent interactions between the N- and C-termini of HD domain. S1 cleavage may occur in the negative regulatory region (NRR), which consists of three LNR and a HD domain.

A pivotal regulatory point in Notch signaling transduction is ligand-induced and metalloprotease-mediated Notch receptor cleavage within the NRR. Signaling is initiated when ligand binding induces metalloprotease cleavage of Notch. Metalloproteinase cleavage site S2 is located within the NRR. NRR is key to prevention of receptor activation in the lack of ligand. Upon reaching the surface of the signal-receiving cell, NEC domains bind to the ligand from the signal-sending cell. The ligand is then endocytosed into the signal-sending cell, where pressure is exerted on Notch, resulting in the dissociation of the Notch heterodimer as well as a second cleavage of Notch by the disintegrin and metalloproteinase family at S2, which is located about 12–13 amino acids outside the transmembrane domain, and the N-terminal cleavage fragment is liganded into the ligand cell. The N-terminal cleavage fragment is then phagocytosed by ligand cells, and the C-terminal cleavage fragment is cleaved a third time by the γ-secretase complex at the S3 to release the soluble NICD. The cleaved NICD is released from the membrane and ectopically translocates to the nucleus, where it combines with signal bind-ing protein for the Mastermind-like family members (MAML1) and other activators to form a Notch transcriptional activator complex that induces the expression of Notch target genes (Pandey et al., 2020).

## Advances in Notch signaling pathway in cancer research

The function of the Notch signaling pathway in the development of tumors has been elucidated, and numerous studies have demonstrated that the dysregulation of the Notch signaling pathway is linked to the onset of various neoplastic diseases, including lung cancer, liver cancer, and colorectal cancer.

### Lung cancer

Notch activity remains a significant contributor to the transition from developmental lung formation to participation in lung plasticity and repair. The peculiar revitalisation of Notch signaling has been demonstrated to be linked to the development and progression of lung cancer, including small cell lung cancer (SCLC) and non-small cell lung cancer (NSCLC). NSCLC is the most common form of lung cancer, constituting for 80%–85% of cases. Notch1 has been implicated in the onset and development of NSCLC and may be useful in assessing disease progression, based on a growing body of evidence (Huang et al., 2020). Notch2 expression levels were approximately 40% higher in patients with advanced NSCLC compared to patients with stage I disease, and the incidence of Notch2 overexpression (22% increase) was significant in patients with disease recurrence (Chen et al., 2017).

The growth, invasion and metastasis of lung cancer were closely related to Notch signaling pathway. The identification of new prognostic biomarkers to guide surveillance was crucial and urgently needed from a clinical perspective. As investigated by Liu et al., the levels of Notch receptors and ligands might be applied as potential markers to assess the prognosis of patients with lung cancer. High levels of Jag1, Dll1, Notch1, and Notch2 mRNA were observed in the better overall survival of the lung adenocarcinoma patients, while higher levels of Jag2, Dll3, and Notch3 mRNA were associated with poor survival (Liu et al., 2016). The opposite prognostic value of Jag1 with Jag2 was attributed to their mutual inhibition and different regulatory mechanisms reported by Choi et al. (2009) They found that the levels of Jag1 and Jag2 were inversely modulated and mutually suppressive. Jag1 levels increased in Jag2 siRNA-transfected cells and Jag2 levels increased in Jag1 siRNA-transfected cells. In comparison to healthy lung tissues, weaker Notch2 expressions existed in NSCLC patients (Baumgart et al., 2015). Yagci et al. (2019) found that the higher susceptibility to lung cancer was notably linked to the G684A and C381T synonymous polymorphisms in the Notch3 gene.

In addition, SCLC accounts for roughly 15% of all lung cancer, which is characterised by highly aggressive, poorly differentiated features (Zhang et al., 2023). George et al. (2015) analysed 71 of 152 fresh-frozen clinical tumour specimens from SCLC patients by genome sequencing, and found the prevalence of Notch mutations in SCLC has been estimated to be in the range of 25%–28%, resulting in a loss of function of the Notch signaling pathway (Ardeshir-Larijani et al., 2018). Specifically, analysis of clinical trial samples confirmed that the Notch inhibitory ligand, DLL3, is expressed in more than 75% of SCLC, and that the majority of SCLC patients have high levels of DLL3 expression (Hu et al., 2022).

### Liver cancer

Liver cancer includes intrahepatic cholangiocarcinoma and hepatocellular carcinoma (HCC), of which HCC is the mainly histological subtype of liver cancer, accounting for more than 90% of the total cases of primary liver cancer. Abnormal Notch signaling is a prominent factor in the progression of HCC tumours. Xie et al. (2021) found that tetraspanin 5 activated Notch signaling by increasing cleavage of the Notch S3 site catalysed by γ-secretase and enhanced Notch-dependent epithelial-mesenchymal transition to promote cell migration and tumour metastasis in HCC. Han et al. (2019) identified mir-449a as a short-term recurrence-associated miRNA that reflects the malignant grade of HCC. It can directly target Notch1 by binding to the 3′UTR of its mRNA to inactivate the Notch signaling pathway, and inhibit the invasion and migration of HCC cells *in vitro* and *in vivo* by regulating epithelial-mesenchymal transition (EMT). Luiken et al. (2020) demonstrated that Notch effectively induces the expression of the target gene HES5 in HCC and has both pro- and anti-tumour effects. In MYC-induced HCC, HES5 inhibited HCC growth, whereas in AKT-induced HCC it promoted tumour formation. As investigated by Nakano et al. (2022), it was demonstrated that two ligands of Notch, Dll4 and Jag1, exhibited mutual antagonism in regulating HCC progression. Dll4 deficiency inactivates the Notch1 signaling pathway and inhibits HCC development. In contrast, knockdown of Jag1 resulted in ectopic expression of Dll4 in otherwise non-expressing hepatocytes with concomitant loss of Notch2 signaling, promoting HCC progression. Dll4 was expressed in cancer cells and engaged Notch1 signalling in an autocrine way, whereas Jag1 was expressed in neighbouring hepatic stellate cells and engaged Notch2 signalling in neighbouring cancer cells.

### Colorectal cancer

Aberrant activation of Notch signaling has been proven to cause colorectal cancer, due to the fact that Notch signaling was vital in maintaining normal intestinal epithelial cells. The Notch signaling pathway was identified to be expressed 10–30 times higher in colon cancer-initiating cells than in widespread used colon cancer cell lines (Sikandar et al., 2010). Furthermore, in a mouse model of colorectal cancer, Notch signaling was abnormally elevated during tumourigenesis, and inhibition of Notch signaling induced adenoma cell differentiation towards goblet cells (Srinivasan et al., 2016). Fazio et al. (2016) revealed that in colorectal cancer cells, inflammation upregulated the Notch1 signaling pathway via metalloproteinase-9, leading to increased invasiveness of intestinal cancer cells. Zheng et al. (2015) performed immunohistochemistry on tumour tissues, paracancerous tissues and distant normal tissues of 47 colorectal cancer patients who did not receive radiotherapy. They found that in comparison to the healthy tissues, Notch1 and Jag1 were overexpressed in cancer tissues, suggesting that Notch1 and Jag1 were essential for the occurrence and development of colorectal cancer, as well as for judging the prognosis. Liao et al. (2018) examined the levels of Notch1 and Jag1 in human colorectal cancer, colorectal cancer adenoma, paracancerous tissues and normal colorectal cancer tissues by immunohistochemistry. The results indicated that the levels of Notch1 and Jag1 was higher in colorectal cancer and colorectal adenoma tissues than in paracancerous tissues and normal colorectal tissues. The silencing of Notch1 in HT29 cells promoted the expression of p21 in HT29 cells, inhibited cell growth, blocked the cell cycle in the G1 phase, and promoted cell apoptosis. Jackstadt et al. (2019) found that a high proportion of human colorectal cancer metastases were strongly positive for Notch1 intracellular structural domain, suggesting that Notch1 signaling is activated in human colorectal cancer metastases and that Notch1 is a key driver of the worst prognostic subtype of colorectal cancer.

## The relationship between Notch mutations and cancer subtypes

Emerging evidences suggested that Notch mutations existed in various cancer subtypes, including lung cancer (Sun et al., 2024; Li et al., 2021; Wang et al., 2023), liver cancer (Shi et al., 2024a), colorectal cancer (Wang et al., 2022), breast cancer (Wang et al., 2015), and so on. Therefore, it was vital to further understand the type and frequency of Notch mutations in the tumor background (Shi et al., 2024b). Mutations in the Notch signaling, including receptor mutations and ligand mutations, were closely associated with a variety of cancer subtypes. Among Notch receptor mutations in SCLC, missense mutations were the most commonly occurring type. Notch1, followed by Notch2, Notch4, and Notch3, was the family member with the highest mutation frequency (Zhang et al., 2023). In terms of the mutations of Notch ligand family in SCLC, it was observed that the overall mutation rate was at a low level of 4%–7%, with members being reciprocally exclusive (Li et al., 2022). Hong et al. (2022) reported that about 25% of patients with SCLC carried mutually exclusive loss-of-function mutations in Notch receptors. As investigated by Almodovar et al., 52% of SCLC displayed inactivating mutations in the Notch family (Almodovar et al., 2018). Notably, the frequency of Notch mutations was considerably reduced in Chinese SCLC patients in comparison with Western SCLC patients (Hu et al., 2019). In comparison to the lung, Notch mutations were less frequent in the liver (Zhang et al., 2023). Su et al. (2018) indicated that Notch1 mutations were associated with poor prognosis in HCC patients, which may be due to disruption of the tumor suppressor effect of Notch1. Initially, active Notch mutations were very rarely seen in the colorectal cancer, but the overexpression of Notch family members (receptors or ligands) were observed (Varga et al., 2020). A recent study suggested that although Notch mutations had no prominent effect on the overall survival of colorectal cancer patients, these mutations could strengthen anti-tumor immunity by modulating the immune microenvironment (Wang et al., 2020). Moreover, Kontomanolis et al. (2018) found that elevated Notch1 activity was associated with mutations in the PEST domain, which was vital for NICD stability of Notch1 in triple negative breast cancers. There were two molecular subtypes of adenoid cystic carcinoma (ACC), ACC-I and ACC-II, of which the enrichment of activated Notch mutations were found in ACC-I (Ferrarotto et al., 2023).

## Strategies for Notch signaling targeting in cancer immunotherapies

Numerous studies have revealed frequent dysregulation of Notch signaling molecule expression in a variety of tumours. Therefore, Therapies targeting the Notch signaling has already found in tumour therapy, which included fission inhibitors such as, Notch ligand-targeting agents, Notch receptor-targeting agents, transcriptional organisers and signaling agonists. γ-secretase inhibitors (GSI) are currently being investigated in preclinical studies as cancer therapeutics and have shown anti-tumour activity in a wide range of tumour types (Song et al., 2023), including non-small cell lung cancer (Liu et al., 2023; Yang et al., 2020), HCC (Cai et al., 2023), breast cancer (Jia et al., 2021; Wang et al., 2024), colorectal cancer and prostate cancer (Federman, 2022).

The GSI is essential for the activation and nuclear translocation of the NICD. CD147 was overexpressed in HCC cells and facilitated cell invasion, migration and proliferation. By binding directly to the NOTCH1 promoter, CD147 was cleaved by γ-secretase and released CD147ICD into the nucleus, where it promoted Notch1 expression. In orthotopic transplantation HCC mouse models, the combined therapy of the GSI and the CD147-targeting antibody showed better efficacy than monotherapy (Yong et al., 2019). GSI was a class of small molecules targeting the Notch signaling pathway, which have been evaluated in pre-clinical and clinical trials for the treatment of NSCLC (Pine, 2018). Das et al. found (Das et al., 2019) a new triazole, NMK-T-057, triggered autophagic cell death in breast cancer cells by preventing γ-secretase-mediated activation of Notch signaling. Downregulation of Notch1 by GSI-I in combination with IL-24 can induce apoptosis and reduce the invasiveness and migratory ability of HepG2 cells (Han et al., 2015). Additionally, GSI increased taxane-induced inhibition of mitosis and apoptosis in colon cancer cells *in vitro* and *in vivo* (Akiyoshi et al., 2008). Cui et al. found that the novel GSI, PF-03084014, may potentiate the anti-tumour effect of doxorubicin in prostate cancer by inhibiting the Notch pathway (Cui et al., 2015).

## Conclusion

Malignant tumours represent a significant global health concern, posing a significant threat to human wellbeing. Abnormalities in Notch signaling disrupt the dynamic equilibrium of Notch signaling-mediated regulatory pathways, ultimately leading to the proliferation of tumour cells. A comprehensive examination of these pathways and their underlying mechanisms may facilitate a deeper understanding of the pathogenesis of malignant tumours. Furthermore, the development of pharmaceutical agents that target different components of the Notch signaling pathway has the potential to impede the progression of malignant tumours, thereby facilitating the creation of more efficacious therapeutic modalities for individuals diagnosed with cancers.
